# Preparation of Cu_2_O-Reduced Graphene Nanocomposite Modified Electrodes towards Ultrasensitive Dopamine Detection

**DOI:** 10.3390/s18010199

**Published:** 2018-01-12

**Authors:** Quanguo He, Jun Liu, Xiaopeng Liu, Guangli Li, Peihong Deng, Jing Liang

**Affiliations:** 1 Hunan Key Laboratory of Biomedical Nanomaterials and Devices, School of Life Science and Chemistry, Hunan University of Technology, Zhuzhou 412007, China; hequanguo@hut.edu.cn (Q.H.); junliu@hut.edu.cn (J.L.); amituo321@163.com (X.L.); liangjingabbey@126.com (J.L.); 2 Department of Chemistry and Material Science, Hengyang Normal University, Hengyang 421008, China

**Keywords:** Cu_2_O nanoparticles, reduced graphene oxide, modified electrode, dopamine detection, electrochemical oxidation

## Abstract

Cu_2_O-reduced graphene oxide nanocomposite (Cu_2_O-RGO) was used to modify glassy carbon electrodes (GCE), and applied for the determination of dopamine (DA). The microstructure of Cu_2_O-RGO nanocomposite material was characterized by scanning electron microscope. Then the electrochemical reduction condition for preparing Cu_2_O-RGO/GCE and experimental conditions for determining DA were further optimized. The electrochemical behaviors of DA on the bare electrode, RGO- and Cu_2_O-RGO-modified electrodes were also investigated using cyclic voltammetry in phosphate-buffered saline solution (PBS, pH 3.5). The results show that the oxidation peaks of ascorbic acid (AA), dopamine (DA), and uric acid (UA) could be well separated and the peak-to-peak separations are 204 mV (AA-DA) and 144 mV (DA-UA), respectively. Moreover, the linear response ranges for the determination of 1 × 10^−8^ mol/L~1 × 10^−6^ mol/L and 1 × 10^−6^ mol/L~8 × 10^−5^ mol/L with the detection limit 6.0 × 10^−9^ mol/L (S/N = 3). The proposed Cu_2_O-RGO/GCE was further applied to the determination of DA in dopamine hydrochloride injections with satisfactory results.

## 1. Introduction

Dopamine (DA), a neurotransmitter secreted in the midbrain region called the substantia nigra of the human body, plays a very important role in the functioning of central nervous, hormone, and cardiovascular system. Generally in brain fluids, DA is present in the 10^−6^ M to 10^−8^ M range, and abnormal levels of DA eventually lead to several neurological disorders, such as Parkinson’s and Schizophrenia diseases [1]. Hence, it is essential to develop a low cost, effective, and sensitive biosensor for detection of DA. It is well known that electrochemical sensors are an excellent technique due to their rapid response, facile operation, sensitivity, and selectivity [2,3]. However, DA in the human body coexists along with ascorbic acid (AA) and urea acid (UA), which act as potential interfering agents [4,5]. It is essential to eliminate the interference of AA and UA in the detection of DA. In recent years, DA biosensors using various nanomaterials have been reported with a satisfactory detection limit [6,7,8,9,10,11,12,13,14]. However, they suffer from various disadvantages, such as cumbersome synthesis, limited sensitivity, and poor selectivity toward DA.

Cu_2_O nanoparticles (Cu_2_O NPs) is a typical *p*-type semiconductor with narrow band gap of 1.9–2.1 eV. The Cu_2_O NPs have been widely used in solar cell, photocatalysis, and sensors due to their excellent catalytic performance [15,16,17]. Cu_2_O NPs-based surfaces have also been applied as efficient CO_2_ electroreduction materials in previous reports [18,19,20]. However, their poor dispersibility is the major obstacle for electrochemical detection with Cu_2_O NPs modified electrodes. Recently, graphenes have widely used in electrochemical sensors attributed to their excellent electrical conductivity, high surface area, and good biocompatibility [21,22,23]. The Cu_2_O/graphene nanocomposites have been developed toward electrochemical detection in recent years. For example, both Zhou and Li groups had prepared the Cu_2_O/graphene nanocomposites modified glassy carbon electrode (GCE), these nanocomposites show the good photocatalytic performance and high selectivity [24,25]. Jiang and coworkers prepared Cu_2_O/N-doped graphene nanocomposite modified GCE for detection of H_2_O_2_. These nanocomposite materials show wider linear response range and lower detection limit [26]. However, Cu_2_O/graphene nanocomposite modified electrodes toward the detection of DA have been scarcely reported. Compared with noble metals (Au and Pt), the low cost and easy preparation of Cu_2_O NPs have gained growing attention in electrocatalytic field. Moreover, reduced graphene oxide (RGO) has been considered as the most promising materials for electrochemical sensing due to their high stability and conductivity among various graphenes. Generally, RGO is synthesized by chemical or hydrothermal reduction of graphene oxide. Compare with these traditional reduction methods, electrochemical reduction is a green and controllable method without using strong reducer [27]. In our previous work, electrochemical reduction was used to prepare the graphene modified acetylene black paste electrode for detection of tryptophan and bisphenol A [28,29,30,31].

Theoretically, the synergistic effect between Cu_2_O NPs and RGO in Cu_2_O-RGO/GCE may endow itself with promising advantages of excellent electrocatalytic activity of Cu_2_O, the large surface area, as well as strong adsorption ability of RGO, and may also improve the selectivity, sensitivity and linear response range of detection of DA. Herein, Cu_2_O-RGO-modified GCEs were fabricated by facile drop-casting followed by the electrochemical reduction method, and the optimum electrochemical reduction conditions for preparing Cu_2_O-RGO/GCE and electrochemical detection conditions for determining DA were also investigated. The morphologies of as-prepared Cu_2_O NPs, RGO and Cu_2_O-RGO nanocomposites were characterized by scanning electron microscope (SEM). Moreover, the electrochemical behavior of DA on the surface of the Cu_2_O-RGO/GCE was studied in detail, and various electrochemical parameters, including pH, scan rate, accumulation potential, and time were discussed carefully. Finally, the Cu_2_O-RGO/GCE was successfully applied in DA detection of real samples.

## 2. Experimental Section

### 2.1. Materials and Chemicals

Graphite powder, sodium nitrate (NaNO_3_), concentrated sulfuric acid (H_2_SO_4_), potassium permanganate (KMnO_4_), hydrogen peroxide (H_2_O_2_), copper sulfate pentahydrate (CuSO_4_·5H_2_O), polyvinylpyrrolidone (PVP), hydrazine hydrate (N_2_H_4_·H_2_O), potassium ferricyanide (K_3_Fe(CN)_6_), potassium ferrocyanide (K_4_Fe(CN)_6_), potassium nitrate (KNO_3_), phosphoric acid (H_3_PO_4_), sodium hydroxide (NaOH), hydrochloric acid (HCl), and ethyl alcohol were purchased from Sinopharm Chemical Reagent Co., Ltd. (Shanghai, China). Dopamine (DA) was purchased from Sigma-Aldrich Co (St. Louis, CA, USA). All these reagents were used as received without further treatment, and ultrapure water was used in all experiments (18.2 MΩ).

### 2.2. Synthesis of Cu_2_O NPs

Fifty milligrams of CuSO_4_·5H_2_O and 24 mg of PVP are dissolved completely in 10 mL of ultrapure water under ultrasound exposure for 30 min. Then, 2 mL of NaOH solution (0.2 M) were added into the above solution. This solution was stirred for 30 min under room temperature, and the blue Cu(OH)_2_ was formed subsequently. Finally, 6 μL of hydrazine hydrate was added under stirring for 20 min in room temperature. The brick red Cu_2_O suspensions were obtained by centrifugation under 5000 rpm. After washing by water and ethyl alcohol repeatedly, the Cu_2_O NPs were prepared as a solution with the concentration of 1 mg/mL.

### 2.3. Synthesis of Cu_2_O-GO Composite Nanomaterials

Graphene oxide (GO) was prepared by modified Hummers’ method [32]. Typically, 23 mL of concentrated H_2_SO_4_ were cooling down to 0 °C, and 0.5 g of graphite powder and 0.5 g of NaNO_3_ were added under mechanical stirring. 3.0 g of KMnO_4_ were added slowly under controlling temperature lower than 5 °C, then the temperature raised to 35 °C under stirring for 2 h to form a mash. Subsequently, 40 mL of water added into the solution slowly under controlling temperature lower than 50 °C, then the temperature was increased to 95 °C for 0.5 h. After adding 100 mL of water, the above solution was added into 20 mL of 30% H_2_O_2_ in batches. The as-obtained golden yellow solution was collected by suction filtration in time, the precipitate was washed by 150 mL of hydrochloric acid (1:10) and 150 mL of H_2_O, respectively. The GO was obtained by drying under 50 °C vacuum overnight. Finally, 100 mg of GO were dispersed in 100 mL of water under ultrasound application for 2 h, the supernatant was obtained with the concentration of 1 mg/mL after centrifugation. 1 mL of Cu_2_O solution (1 mg/mL) was added 20 mL of GO solution under ultrasound for 2 h, and the Cu_2_O-GO composite nanocomposites were obtained.

### 2.4. Fabrication of Cu_2_O-RGO-Modified GCE

Firstly, the GCE was polished by α-Al_2_O_3_ with different fine sizes (1.0 μm, 0.3 μm, and 0.05 μm), then was immersed in ethyl alcohol and water under ultrasound application for 1 min, respectively. The Cu_2_O-RGO/GCEs were fabricated via drop-casting of the Cu_2_O-RGO dispersion on the GCE, followed by an electrochemical reduction process. For comparison, reduced graphene oxide-modified GCEs (RGO/GCE) were also prepared similarly.

### 2.5. Characterization

Scanning electron microscopy (SEM, Hitachi S-3000N, Tokyo, Japan) was used to photograph SEM images at 30 kV. The electrochemical behaviors of as-prepared samples were tested by electrochemical workstation (CHI660E, Shanghai Chenhua Instrument Co. LTD., Shanghai, China) and Polarographic Analyzer (JP-303E, Chengdu Instrument Factory, Chengdu, China).

### 2.6. Electrochemical Experiments

All electrochemical experiments including cyclic voltammetry (CV), second-order derivative linear sweep voltammetry (SDLSV), and electrochemical impedance spectroscopy (EIS) were carried out with a standard three-electrode system, using bare or modified GCEs, platinum wire electrode, and saturated calomel electrode (SCE) as working counter, counter electrode and reference electrodes, respectively. The electrochemical response was performed using CV on Cu_2_O-RGO/GCE in a freshly prepared 0.1 M PBS containing 1 × 10^−5^ mol/L DA. The EIS was measured at their open circuit voltage with 5 mV amplitude, using 5 × 10^−3^ mol/L [Fe(CN)_6_]^3−/4−^ as redox probe solution. The frequency ranged from 1 × 10^5^ Hz to 0.1 Hz. The sensing performance of DA on Cu_2_O-RGO/GCE was investigated using SDLSV in a 10 mL electrochemical cell containing 0.1 M PBS. Both the CVs and SDLSV were recorded at a scan rate of 100 mV/s, after a suitable accumulation period under stirring at 500 rpm and a 5 s rest. The potential scan ranges were −0.2 to 1.0 V for CV and 0–1.1 V for the SDLSV. The CV was measured by CHI 660E electrochemical workstation (Chenhua Instrument Co. Ltd., Shanghai, China), and SDLSV was recorded by a JP-303E Polarographic Analyzer (Chengdu Instrument Company, Chengdu, China).

### 2.7. Analysis of Real Samples

Dopamine hydrochloride injection samples were purchase from Aladdin Reagent Co. (Shanghai, China). Two milliliter dopamine hydrochloride injections (containing dopamine hydrochloride 2 mg) were diluted to 100 mL with 0.1 M PBS (pH = 3.5) to obtain DA diluent. Then dopamine hydrochloride injection samples with various concentration were prepared by adding a certain amount of DA diluent and diluting with 0.1 M PBS (pH = 3.5) in a 10 mL volumetric flask. The content of dopamine in the dopamine hydrochloride injections was measured using SDLSV by the standard addition method under the optimal detection conditions.

## 3. Result and Discussion

### 3.1. Morphologic Characterization of Cu_2_O-GO Nanocomposites

**Figure 1:**
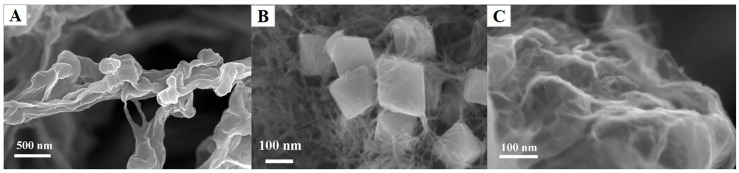
SEM images of RGO (**A**), Cu_2_O (**B**) and Cu_2_O-RGO composite nanoparticles (**C**).

The SEM images of these as-prepared RGO, Cu_2_O and Cu_2_O-RGO are depicted in Figure 1A–C, respectively. As shown in Figure 1A, the RGO nanosheets with plicated surface are evident, indicating that the RGO is synthesized successfully. The SEM image of Cu_2_O nanoparticles is presented in Figure 1B, the octahedron shape of Cu_2_O with uniform size is observed. Figure 1C shows the SEM image of Cu_2_O-RGO nanocomposites, where the Cu_2_O NPs are coated with RGO nanosheets, indicating that the Cu_2_O NPs are well combined with RGO.

### 3.2. Electrochemical Characterization

**Figure 2:**
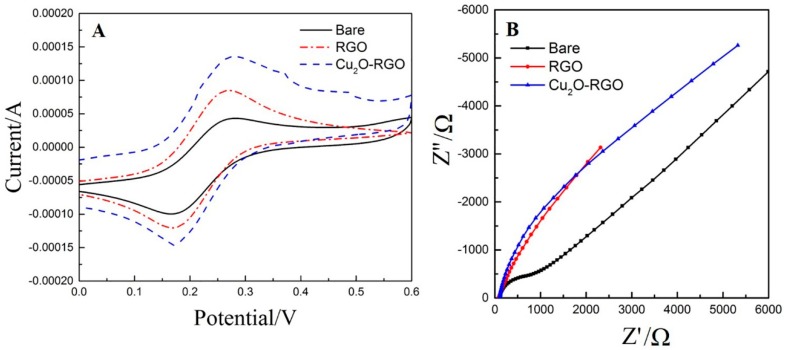
Cyclic voltammograms (**A**) and Nyquist plots (**B**) of bare GCE, RGO, or Cu_2_O-RGO-modified GCEs in 5 × 10^−3^ mol/L [Fe(CN)_6_]^3−/4−^ solution. The CVs was recorded in 0.1 M PBS (pH = 3.5) at the scan rate of 100 mV/s. The Nyquist plots was measured with alternating current (AC) amplitude of 5 mV, from 1 × 10^5^ Hz to 0.1 Hz at their open circuit voltage.

The CV curves of bare or modified GCEs recorded in 5 × 10^−3^ mol/L of [Fe(CN)_6_]^3−/4−^ solution are presented in Figure 2A. The reduction peak currents of bare GCE, RGO/GCE and Cu_2_O-RGO/GCE are 8.808 × 10^−5^ A, 1.187 × 10^−4^ A, and 1.311 × 10^−5^ A, respectively. According to Randles-Sevcik equation, the electrochemical active area of bare GCE, RGO/GCE, and Cu_2_O-RGO/GCE were calculated as 0.075 cm^2^, 0.101 cm^2^, and 0.112 cm^2^, respectively. The electrochemical active area of bare GCE coincides with the geometric area (Φ 3.0 mm, 0.071 cm^2^), and the electrochemical active area of RGO/GCE and Cu_2_O-RGO/GCE are 1.3 and 1.5 times of that of bare GCE. This phenomenon is probably related to the large specific surface area of Cu_2_O and RGO. The increase of electrochemical active area could not only improve the adsorption capacity of DA, but also increase the catalytic sites for DA oxidation. As a result, the electrochemical oxidation of DA was accelerated greatly. Additionally, the electrode interface property is also investigated by electrochemical impedance spectroscopy (EIS), and the results are presented in Figure 2B. The radius of the semicircle in Nyquist plot represent the charge transfer resistance (*R_ct_*). The *R_ct_* of RGO/CCE is larger than that of Cu_2_O-RGO/GCE, because the electrical conductivity of Cu_2_O NPs is poor due to their semiconductive property. However, the *R_ct_* of both RGO and Cu_2_O-RGO nanocomposite-modified electrodes are much lower than that of bare GCE because of the high conductivity of RGO.

### 3.3. Optimization of Electrochemical Reduction Condition

**Figure 3:**
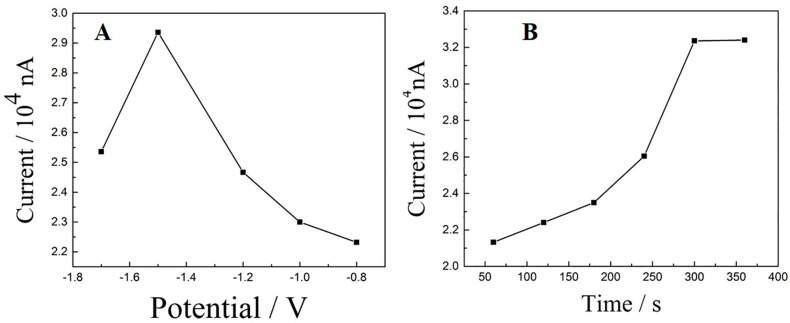
Optimization of reduction potential (**A**) and reduction time (**B**) for electrochemical reduction of Cu_2_O-GO nanocomposites.

In order to seek optimum preparation conditions for Cu_2_O-RGO/GCE, the electrochemical conditions, including reduction potential, as well as time were further investigated. Generally, the potential range for electrochemical reduction of GO is −1.5V to −1.0 V. In this study, Cu_2_O-RGO/GCE samples were prepared from Cu_2_O-GO/GCE by potentiostatic method under various reduction potentials (−1.7 V, −1.5 V, −1.2 V, −1.0 V, and −0.8 V). After reduction for 300 s, the as-prepared Cu_2_O-RGO/GCEs were used for the detection of DA (1 × 10^−5^ mol/L). As shown in Figure 3A, the largest oxidation peaks current (*i_pa_*) is obtained when the reduction potential is −1.5 V. Furthermore, the oxidation peaks of various Cu_2_O-RGO/GCEs fabricated with different reduction time (60 s, 120 s, 180 s, 240 s, 300 s, and 360 s) are also compared, while the reduction potential was fixed as −1.5 V. As shown in Figure 3B, the oxidation peak currents *i_pa_* increase gradually when the reduction time increases from 60 s to 300 s, the maximum *i_pa_* is obtained in 300 s. Afterwards, *i_pa_* remains stable with prolonging the reduction time. As a result, the reduction potential and time for Cu_2_O-RGO/GCE preparation are suggested as −1.5 V and 300 s, respectively.

### 3.4. The DA sensing of Modified Electrodes

**Figure 4:**
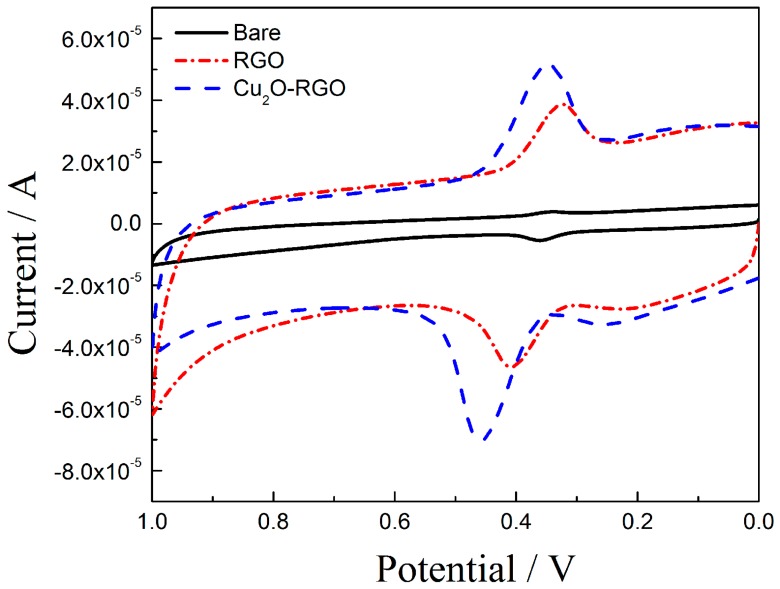
Cyclic voltamogramms obtain for 1 × 10^−5^ mol/L dopamine on bare GCE, RGO/GCE, and Cu_2_O-RGO/GCE in the presence of 0.1 M PBS (pH = 3.5) as supporting electrolyte. Scan rate: 0.1 V/s.

The DA sensing of GCE, Cu_2_O/GCE, and Cu_2_O-RGO/GCE was investigated using CV recorded at 100 mV/s in the 0.1 M of PBS, and the results are shown in Figure 4. On the bare GCE, both oxidation peak current (*i_pa_* = 5.415 μA) and reduction peak current (*i_pc_* = 3.899 μA) is very low, indicating a poor electrochemical response of bare electrode. On the RGO/GCE, the *i_pa_* and *i_pc_* is improved to 36.565 μA and 31.149 μA, respectively. This phenomenon is probably due to the excellent electrical conductivity, large surface area of RGO, and great adsorption capacity of DA. The strong π–π interactions between the phenyl ring of DA and the two dimensional planar carbon structure of RGO is beneficial to increasing the adsorption capacity of DA. Moreover, when Cu_2_O-RGO/GCE was used as work electrode, the *i_pa_* increases to 70.720 μA and *i_pc_* increases to 51.558 μA. The peak currents enhanced more distinctly than those of bare GCE and RGO/GCE because of the synergistic effect of Cu_2_O NPs and RGO combination. Specifically, the RGO with large surface area could increase the adsorption capacity of DA. On the other hand, the electrocatalytic properties of Cu_2_O NPs could accelerate the electron transfer between Cu^+^ and Cu^2+^, and then increase the response current density [33]. Thus, the as-prepared Cu_2_O-RGO/GCE could be used to detect the DA effectively.

### 3.5. Optimization of the Detection Condition of DA

#### 3.5.1. The Influence of pH

**Figure 5:**
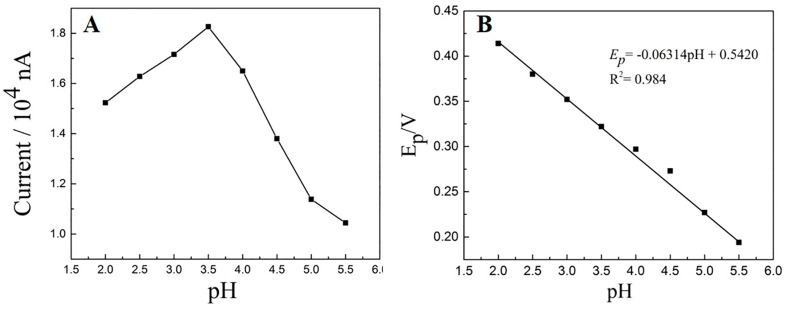
(**A**) The effect of pH on the oxidation peak current of 1 × 10^−5^ mol/L DA; and (**B**) the linear relationship between oxide peak potential and pH.

The oxidation of DA dependents strongly on pH of medium, so it is well worth optimizing the pH for DA detection. The electrochemical responses of DA were investigated in PBS under different pH (2.0–5.5). The CV curves under different pH values are presented in Figure 5A. At a pH of 3.5, the maximum *i_pa_* of DA is obtained. Moreover, the linear relationship between oxidation peak potential *E_p_* and pH is evidently observed at the pH range from 2.0 to 5.5. As shown in Figure 5B, the linear equation is *E_p_* = −0.06314 pH + 0.542 (R^2^ = 0.984), and the slope is −63 mV/pH, highly closing to theoretical value (−59 mV/pH). It implies that the number of electron and proton participated in electrochemical oxidation process is the same [34].

#### 3.5.2. Effect of Accumulation Conditions

**Figure 6:**
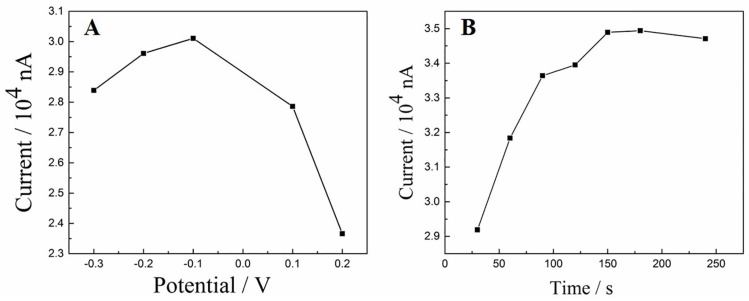
The effect of accumulation potential (**A**) and accumulation time (**B**) on the oxidation peak current of 1 × 10^−5^ mol/L DA.

The effect of accumulation potential as well as time on the oxidation current of DA obtained at the Cu_2_O-RGO/GCE were investigated, because the accumulation step is usually a simple and effective method to improve the sensitivity. The oxidation peak currents of 1 × 10^−5^ mol/L DA were measured after accumulation process at different accumulation potentials (−0.3 to 0.2 V) for 240 s. As shown in Figure 6A, the largest oxidation peak current appeared at the accumulation potential of −0.1 V, indicating that −0.1 V is the optimal accumulation potential. Afterwards, the various accumulation time is also investigated while the accumulation potential was fixed as −0.1 V. The relationship of accumulation time and oxidation peak current is presented in Figure 6B. With prolonging the accumulation time, the oxidation peak currents increase rapidly in the first 150 s. Afterwards the oxidation peaks current keep stable with further increase of accumulation time. This phenomenon could be ascribed to the saturated adsorption of DA on the electrode surface. Thus, the accumulation time is chosen as 150 s.

#### 3.5.3. The Influence of Scan Rate

**Figure 7:**
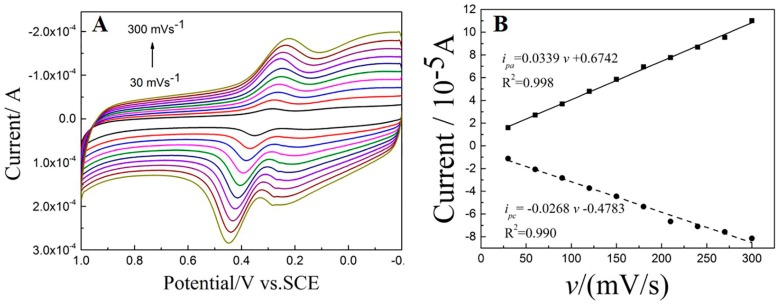
The effect of scan rate (*v*) on the peak current of 1 × 10^−5^ mol/L DA. (**A**) CVs of 1 × 10^−5^ mol/L DA on the Cu_2_O-RGO/GCE recorded in 0.1 M PBS with different scan rates (*v*); and (**B**) linear relationship between peak currents and scan rate (*v*).

The scan rate is an important parameter that influences the electrochemical response of DA. The electrochemical behaviors were investigated using CV in 1 × 10^−5^ mol/L of DA in PBS (0.1 M, pH = 3.5) under different scan rate (30~300 mV/s), and the results are presented in Figure 7A. With the increase of scan rate, both oxidation and reduction peak currents increase evidently. It is noteworthy that the background currents also increase. Figure 7B show the linear relationship between redox peak currents (*i_pa_* and *i_pc_*) and scan rate (*v*), corresponding linear equations are *i_pa_* = −0.0268 *v* − 0.4783 (R^2^ = 0.990) and *i_pc_* = 0.0339 *v* + 0.6742 (R^2^ = 0.998), respectively. These results suggest that the electrochemical oxidation of DA on the Cu_2_O-RGO/GCE is an adsorption-controlled process. Thus, accumulation method is applied for increasing the response currents density in subsequent experiment. Although the currents increase with the scan rate rising, the background currents are also improved. Thus, a suitable scan rate is advisable as 100 mV/s for enhancing signal to noise ratio (SNR) and reducing the background current at the same time. Moreover, with the increasing of scan rate, oxidation peak current is shifted positively, and reduction peak current is shifted to negative direction in contrast. This demonstrates that the DA oxidation is a quasi-reversible reaction.

### 3.6. Interference Studies

**Figure 8:**
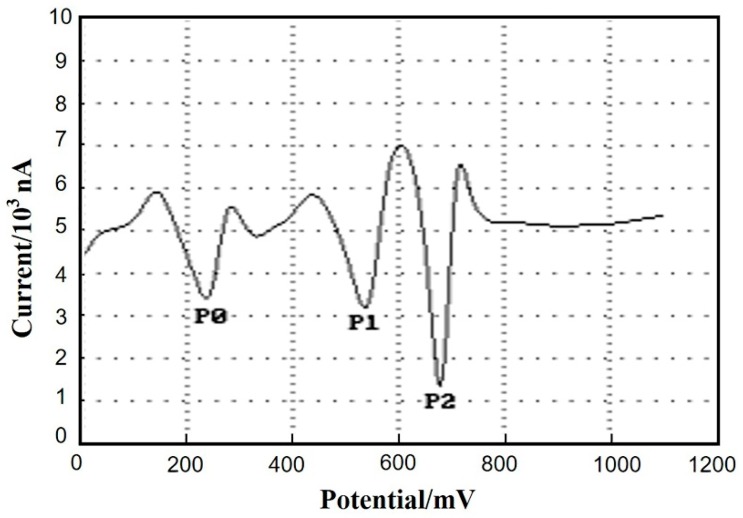
The SDLSV of DA (1 × 10^−5^ mol/L) on the Cu_2_O-RGO/GCE in the presence of AA (1 × 10^−5^ mol/L), and UA (1 × 10^−5^ mol/L). P_0_, P_1_, and P_2_ denotes the peak potentials of AA, DA, and UA, respectively. Scan potential range: 0~1.1 V; scan rate: 100 mV/s; supporting electrolytes: 0.1 M PBS.

As is well known, the DA detection is seriously interfered by AA and UA in human body, since AA and UA often coexist simultaneously in the human body and their response peaks overlap easily. The current responses of DA (2 × 10^−5^ mol/L), AA (1 × 10^−5^ mol/L), and UA (1 × 10^−5^ mol/L) are investigated by SDLSV method in this section. Considering its high resolution and good sensitivity, the SDLSV technique was used for simultaneous detection of DA, AA, and UA. As shown in Figure 8, the oxidation peak currents of AA, DA, and UA are separated each other evidently. P_0_, P_1_, and P_2_ are the peak potentials of AA, DA, and UA, respectively. The potential difference (∆Ep) between AA and DA is 204 mV, and the ∆Ep between DA and UA is 144 mV. Moreover, the intensity of oxidation peak current of DA is still prominent and stable even under the interference of AA and UA. This result indicates that the proposed Cu_2_O-RGO/GCEs possess good selectivity and anti-interference property due to the synergistic effects of Cu_2_O-RGO nanocomposities.

### 3.7. Calibration Curve and Detection Limit 

**Figure 9:**
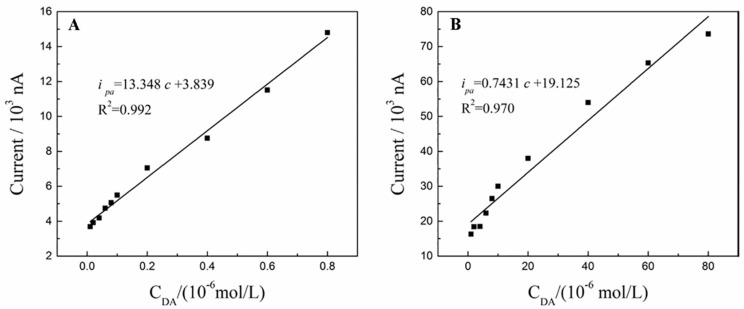
The linear relationship between the oxidation peak *i_pa_* and the concentration of DA in the range of 1 × 10^−8^ mol/L~1 × 10^−6^ mol/L (**A**) and 1 × 10^−6^ mol/L~8 × 10^−5^ mol/L (**B**).

The quantitative analysis of DA is carried out under the optimal detection conditions. The oxidation peak currents *i_pa_* increase as the concentration of DA increases from 1 × 10^−8^ mol/L to 1 × 10^−6^ mol/L, and the linear relationship between oxidation peak currents *i_pa_* and the concentration of DA is obtained as *i_pa_* (10^3^ nA) = 13.348 *c* + 3.839 (R^2^ = 0.992) (Figure 9A). Furthermore, when the concentration of DA ranges from 1 × 10^−6^ mol/L to 8 × 10^−5^ mol/L, another linear relationship between oxidation peak currents *i_pa_* and the concentration of DA is also obtained as *i_pa_* = 0.7431 *c* + 19.125 (R^2^ = 0.970) (Figure 9B). *i_pa_* is oxidation peak currents, and the unit is 10^3^ nA. *c* is the concentration of DA, and the unit is 10^−6^ mol/L. The detection limit (S/N = 3) is estimated as 6.0 × 10^−9^ mol/L. The wider linear range and lower detection limit are obtained as compared with previous literature reports [35,36,37,38,39,40] as summarized in Table 1.

### 3.8. Practical Applications

SDLSV is an extensively used electrochemical technique for biomolecules detection due to its high resolution and sensitivity. Thus, the dopamine hydrochloride injection sample with various concentration was measured by using SDLSV under the optimal conditions. The results of these detections are listed in Table 2, the detection values of DA are well consistent with standard values, and the RSD is −3.20~1.12%. The recovery rate is 96.5~104.4%. These results suggest that the Cu_2_O-RGO/GCE could be used for DA detection of real samples.

## 4. Conclusions

In summary, the proposed Cu_2_O-RGO/GCE are successfully used for DA detection. The optimal reduction conditions for the Cu_2_O-RGO/GCE fabrication are as follows: reduction potential is −1.5 V, and reduction time is 120 s. After the electrochemical reduction, the Cu_2_O NPs is observed with well-coated RGO. Moreover, the electrochemical oxidation process of DA occurred on the Cu_2_O-RGO/GCE is an adsorption-controlled process. The oxidation peaks of AA, DA, and UA are well separated, suggesting high selectivity for DA detection. The Cu_2_O-RGO/GCE have wide linear range (1 × 10^−8^ mol/L~1 × 10^−6^ mol/L and 1 × 10^−6^ mol/L~8 × 10^−5^ mol/L), and a low detection limit (S/N = 3) of 6.0 × 10^−9^ mol/L. Finally, these modified GCE are successfully used for detection of DA in dopamine hydrochloride injections. The facile fabrication in conjunction with rapid response, the low detection limit, and the wide linear range for DA sensing is the advantage of this paper.

## Figures and Tables

**Table 1:** Comparison the determination of DA between Cu_2_O-RGO/GCE and modified electrodes reported in the literature.

Modified Electrodes	Linear Range (M)	Detection Limit (M)
Cu_2_O-RGO/GCE	1 × 10^−8^~1 × 10^−6^; 1 × 10^−6^~8 × 10^−5^	6.0 × 10^−9^
Fe_3_O_4_@Au-Gr/GCE [35]	5 × 10^−7^~5 × 10^−5^	6.5 × 10^−7^
Fe_3_O_4_-RGO/CPE [36]	2 × 10^−8^~5.8 × 10^−6^	6.5 × 10^−9^
Mn_3_O_4_-RGO/GCE [37]	1 × 10^−6^~1.45 × 10^−3^	2.5 × 10^−7^
MnO_2_ NR-RGO/GCE [38]	5 × 10^−8^~4 × 10^−4^	1.0 × 10^−8^
NiO-RGO/GCE [39]	5 × 10^−7^~3.2 × 10^−5^	3.8 × 10^−8^
ZnO NR-RGO/Graphite [40]	5 × 10^−7^~1 × 10^−4^	2.5 × 10^−7^

**Table 2:** The results of determination of dopamine hydrochloride injections (*n* = 4).

No.	Standard Value (μM)	Determination Value (μM)	Added (μM)	Total Found	Recovery (%)	RSD (%)
1	13.14	12.72	10.00	23.16	104.4	−3.20
2	27.63	27.94	30.00	56.91	96.5	1.12
3	48.62	47.38	50.00	96.77	98.8	−2.25
